# Place of death in children and young people with cancer and implications for end of life care: a population-based study in England, 1993–2014

**DOI:** 10.1186/s12885-016-2695-1

**Published:** 2016-09-19

**Authors:** Wei Gao, Julia Verne, Janet Peacock, Charles Stiller, Claudia Wells, Anne Greenough, Irene J. Higginson

**Affiliations:** 1 King’s College London, Cicely Saunders Institute, Department of Palliative Care, Policy and Rehabilitation, Bessemer Road, Denmark Hill, London, SE5 9PJ UK; 2 Public Health England, Knowledge & Intelligence Team (South West), Grosvenor House, 149 Whiteladies Road, Bristol, BS8 2RA UK; 3 King’s College London, Division of Health and Social Care Research, Addison House, Guy’s Campus, London, SE1 1UL UK; 4 Public Health England, Childhood Cancer, 4150 Chancellor Court, Oxford Business Park South, Oxford, OX4 2GX UK; 5 Office for National Statistics, Life Events and Population Sources Division, Cardiff Road, Newport, Wales NP10 8XG UK; 6 King’s College London, School of Medicine, Division of Asthma, Allergy and Lung Biology, Denmark Hill, London, SE5 9RS UK

**Keywords:** End of life care, Children and young people, Place of death, Cancer, Palliative care, Inequality

## Abstract

**Background:**

Efforts to improve end of life care (EoLC) have made tangible impacts on care in adults, including enabling more people to die at their preferred place of death (PoD), usually home or hospices. Little is known how the PoD in children and young people (CYP, ≤24 years) has changed over time, especially in the context of a series of national initiatives for EoLC improvement since the late 1990s. To inform evidence-based policy-making and service development, we evaluated the national trends of PoD and the associated factors in CYP who died with cancer.

**Methods:**

Population-based observational study in the National Health Service (NHS) England, 1993-2014. All non-accidental CYP deaths with cancer (*N* = 12,774) were extracted from the death registration database of the Office for National Statistics (ONS).

**Results:**

Hospital deaths reduced from >50 to 45 %, hospice deaths were rare but more than doubled from 6 % in 1993–2000 to 13 % in 2005–2014, and home deaths fluctuated at around 40 %. Those aged 0–19 years were more likely to die at home than young adults (adjusted proportion ratio (PRs): 1.23–1.62); haematological cancer patients or those with 2+ comorbid conditions had higher chances of hospital death (PRs for home: 0.18–0.75, hospice: 0.04–0.37); deprivation was associated with a reduced chance of home death (PRs: 0.76–0.84). The residential region affected hospice but not home deaths. The variations of PoD by cause of death, comorbid conditions and deprivation slightly decreased with time.

**Conclusions:**

Hospitals and home were the main EoLC settings for CYP with cancer. Home death rates barely changed in the past two decades; deaths in hospitals remained the most common but slightly shifted towards hospices. CYP with haematological malignancy or with comorbid conditions had persistently high hospital deaths; these cases had an even lower chance of deaths in hospices (50 %) than at home. There were deprivation- and area-related inequalities in PoD which may need service- and/or policy-level intervention. The findings highlight a need for CYP specific initiatives to enhance EoLC support and capacities both at home and in hospices.

## Background

A “good death” as understood by many is one that occurs at home or in a home-like environment (e.g., hospice) [1]. However, when it is weighted against the basic physical needs, which are free of pain and other symptoms, to die in one’s preferred place of death (PoD) becomes far less important [2]. Children and young people (CYP) with cancer and their carers also rate the “physical comfort” as the top concern [3, 4]. Quality end of life care (EoLC) encompasses four dimensions of needs: physical, psychological, social and spiritual, and should also extend the support to family members throughout the dying process [5]. Unless these needs, particular the symptom management, can be addressed at a consistently high standard across various care settings, patients and their carers cannot meaningfully exercise their choice for PoD. In fact, most deaths are still happening in the least preferred option – hospital [6–9]. In this context, the PoD remains a useful indicator for population-based EoLC needs and how the needs have been met.

A series of EoLC tools have been developed and sequentially implemented in the United Kingdom since late 1990s, including the Gold Standard Framework, Preferred Priorities for Care and the now phased out Liverpool Care Pathway for the Dying Patient [10–12]. The NHS End of Life Care Programme launched at the end of 2004 has contributed significantly to the roll out of these programmes [1, 13]. Although primarily targeted for adults, these tools have also been adapted for use in CYP. From 2004, framework and strategic documents specifically designed for CYP were developed, in an attempt to raise the quality standard of EoLC [14, 15]. At around the same time, paediatric palliative care services in England received its largest ever single investment (£48 millions) from the Big Lottery Fund towards their nationwide development, aiming to increase the provision of, and access to, hospice and community-based support to children with cancer and other life-threatening and life-limiting conditions [16]. Recent studies in England and in the States found a PoD shift from hospitals to either home or hospices among adults who died from cancer or dementia, corresponding with the national EoLC improvement initiatives [7, 17, 18].

While to some extent overlapping with adults, CYP have their distinct care needs and characteristics [19–21]. It is essential to understand whether the primarily adult-focused national efforts worked for CYP and how. It is crucial for policy and service development. Two studies examined the population-based PoD patterns in CYP and found significant variation, but neither of them did so in the changing context [22, 23.] This study aimed to evaluate how the PoD and the determinants in CYP with cancer changed over time. We chose to focus on cancer as it is a leading non-accidental mortality in CYP [24–26], but not currently optimally served by palliative and EoLC service [19, 21].

## Methods

### Study design and setting

A population-based study in the National Health Service (NHS) England.

### Data sources and study populations

Data were collected by the Office for National Statistics (ONS) from all death registrations in England, 1993–2014. By law in England, a death must be registered within 5 days, unless it becomes the subject of a coroner’s inquiry. The underlying cause of death (CoD) was recorded in the database using the 9^th^ (1993–2000) or 10^th^ (2001–2014) edition of International Classification of Diseases (ICD-9, ICD-10) codes. All non-external cause of deaths (ICD-9: E codes; ICD-10: T79-T98, Y35–36 & Y40–Y98) that occurred before the age of 24 years, with cancer as the underlying or contributing CoD (ICD-10: C00–C97; ICD-9: 140–209), were included for this study.

### Variables

The study outcome – PoD, was grouped into four categories: hospital, home, hospice, and elsewhere. Hospice, in this context, refers to a dedicated unit with in-patient beds, staffed by specialists in palliative care, and is usually freestanding from hospitals. Admission to hospice is not restricted to those at the EoL, CYP are often admitted for symptom control, or other reasons (e.g., respite care). Hospices are not for profit and do not charge for admission. Explanatory variables included: age at death (<1, 1–4, 5–9, 10–14, 15–19, 20–24), gender (male, female), the underlying cause of death (See Table 1 for ICD-9/10 codes) – the number of contributing cause of deaths (defined as diseases or injuries that contributed to the fatal outcome), year of death, the index of deprivation, the rural/urban indicator and the region (defined by Clinical Senate, 2013) [27] of the deceased residential address. Age was analysed as an ordered categorical variable rather than a continuous variable to facilitate interpretation and comparison with the other studies. The deprivation - an indicator of socioeconomic position [28] - was measured by the lower super output area (LSOA) quintile of the income deprivation affecting children index (IDACI), where 1 = most deprived and 5 = least deprived. The IDACI is calculated by the Office of the Deputy Prime Minister and measures in a local area the proportion of children under the age of 16 that live in low income households. The local areas for which the index is calculated are LSOA. A LSOA is a low-level geographic area that is designed for reporting small area statistics in England and Wales. There are 32,482 LSOAs in England; each area has a minimum population size of 1,000 and an average of 1,500. The rural/urban settlement was classified using the 2011 Census data at the level of LSOA. We used IDACI 2001, IDACI 2004 and IDACI 2007 to map the residential area-based deprivation of the deceased for the period 1993–2000, 2001–2004, 2005–2014, respectively. The study period was divided into three intervals in order to examine changing patterns. The division took into consideration the launch, implementation and roll-out of several national initiatives around 2004/2005 for improving EoLC [1, 13]; and also the ONS’s ICD coding system changing from the ICD-9 to the ICD-10 in 2001.

**Table 1:** ICD-9 & 10 codes for underlying causes of death classification

Group	Underlying cause of death*	ICD-10 codes	ICD-9 codes
1	Leukaemia: ALL	C91.0	204.0
2	Leukaemia: AML	C92.0, C92.4, C92.5, C92.6, C92.8, C93.0, C94.0, C94.2	205.0, 206.0, 207.0, 207.2
3	Leukaemia: other	C91-C95 excluding above	204–208 excluding above
4	Lymphomas: Hodgkin’s	C81	201
5	Lymphomas: non-Hodgkin’s	C82-C86	200, 202
6	Myeloma	C90	203
7	Brain, other CNS & Intracranial tumours	C70-C72, C75.1-C75.3, D32-D33, D35.2-D35.4, D42-D43, D44.3-D44.5	191–192, 194.3–194.4, 225, 227.3–227.4, 237.0, 237.1, 237.5, 237.6, 239.6
8	Connective tissue cancer	C46, C47, C49	171
9	Bone sarcoma	C40-C41	170
10	Renal tumours	C64	189.0
11	Hepatic tumours	C22	155
12	Adrenal tumors including neuroblastoma	C74	194.0
13	Retinoblastomas	C69.2	190.5
14	Other malignant	C00-C97 excluding all above C codes	140–209 excluding above
15	Other neoplasm	D00-D48 excluding above D codes	210–239 excluding above
16	Non-cancer	A00-B99, D50-R99	000–139, 240–799

*Underlying causes of death grouping: Leukemia: 1–3; Lymphomas & other haematological: 4–6; Brain & other CNS tumors: 7; Bone & connective tissue: 8–9; Renal/Liver/Adrenal including neuroblastoma: 10–12; Other malignant: 13–15; Non-cancer: 16

### Statistical analysis

Data was first checked for errors and missing values; if the missing data was less than 5 %, the records were deleted using the list-wise approach [29]. Data was described using frequency, proportion and 95 % confidence interval (CI). The time trend in proportion of CYP cancer deaths in four PoDs was evaluated and tested using Tobit regression models, weighted by the total number of CYP deaths and the number of CYP cancer deaths. The proportion was age- and sex-standardised using the 1993’s structure. The year of death was treated as a continuous variable. The regional variation was visualised with geographical maps.

Modified Poisson regression models were used to evaluate the factors independently associated with PoD. Two sets of models were constructed separately for the three time periods: home (1) versus hospital (0), and hospice (1) versus hospital (0). All explanatory variables were kept in the model. To ensure statistical efficiency, we categorised age into three groups: 0–14, 15–19, 20–24. The strength of association was measured by the proportion ratio (PR) – a measure of relative risk (RR), estimated from the period-specific models.

Possible two-way interaction effect between factors were explored and tested, using the likelihood ratio test of nested reduced models with and without the interaction terms. The collinearity was evaluated with the condition index (CI). A CI value greater than 30 indicates the presence of collinearity. If two factors were found significantly associated with each other, sensitivity analysis would be carried out by running separate models omitting one of the concerned variables. The resulting parameter estimates would then be compared to those from the main analysis models.

All statistical tests were two-sided; statistical significance is defined as *p* < 0.05. All analyses were performed using the SAS 9.4 (SAS Institute Inc, Cary, North Carolina). The GIS mapping was completed with R version 3.1.2 software (www.r-project.org) and R Studio Version 0.98.1091.

## Results

### Demographic and clinical details, information on potential confounders

**Fig. 1:**
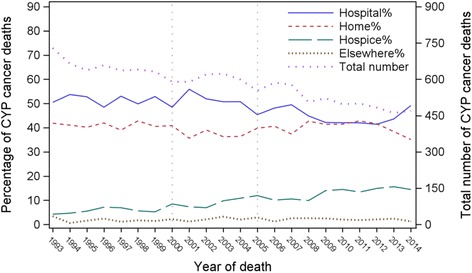
Proportion of place of death and total number of deaths in children and young people who died with cancer, England 1993–2014

Between 1993 and 2014 in England, 12,774 CYP died with cancer as an underlying or contributory cause of death, accounting for 18.7 % of non-accidental causes of CYP deaths. The proportion of cancer deaths increased from 18 to 25 %. The number of CYP cancer deaths fell 36 % from 729 in 1993 to 470 in 2014 (Fig. 1). Table 2 shows the demographic and clinical characteristics of the study population. Proportions of cancer deaths by age group did not vary much over time, and half died aged between 15–24 years. More males than females died from cancer. Haematological malignancies (Leukaemia, Lymphoma & other haematological cancers, *N* = 2580) comprised over 30 % of CYP cancer deaths prior to 2005, but its proportion reduced over time to the same level as brain & CNS cancer (27 %, *N* = 1369) in 2005–2014. A small proportion (3.2–4.2 %) of CYP with cancer died from a non-cancer cause, nearly half (45 %) of them had a haematological cancer mentioned on their death certificates. Around 10 % of CYP cancer deaths had three or more contributing causes of death. There was a small proportion increase of CYP deaths with cancer, living in the most deprived area (44 to 46 %). Most CYP dying with cancer (~84 %) lived in an urban area (Table 2).

**Table 2:** Demographical and clinical characteristics of children and young people who died with cancer, England 1993–2014 (*N* = 12,774)

Variable	Value	Year of death		
		1993–2000	2001–2004	2005–2014
Total	–	5187	2431	5156
Average/year	–	649	608	517
Age	<1	162(3.1)	63(2.6)	152(2.9)
	01–04	736(14.2)	325(13.4)	722(14.0)
	05–09	887(17.1)	377(15.5)	785(15.2)
	10–14	808(15.6)	411(16.9)	743(14.4)
	15–19	1062(20.5)	574(23.6)	1169(22.7)
	20–24	1532(29.5)	681(28.0)	1585(30.7)
Gender	Female	2193(42.3)	1059(43.6)	2244(43.5)
	Male	2994(57.7)	1372(56.4)	2912(56.5)
Underlying cause of death	Leukaemia	1366(26.3)	564(23.2)	992(19.2)
	Lymphoma & other haem-atological cancers	447(8.6)	203(8.4)	377(7.3)
	Bone & connective	694(13.4)	384(15.8)	812(15.7)
	Brain & CNS etc.	1117(21.5)	580(23.9)	1384(26.8)
	Renal/Liver/Adrenal including neuroblastoma	467(9.0)	226(9.3)	498(9.7)
	Other malignant & neoplasm	876(16.9)	389(16.0)	928(18.0)
	Non-cancer	220(4.2)	85(3.5)	165(3.2)
N. contributing CoDs	0	2476(47.7)	1209(49.7)	2757(53.5)
	1	1703(32.8)	680(28.0)	1300(25.2)
	2	648(12.5)	311(12.8)	605(11.7)
	3+	360(6.9)	231(9.5)	494(9.6)
IDACI quintile	Most deprived	1252(24.1)	585(24.1)	1317(25.5)
	2	1033(19.9)	494(20.3)	1064(20.6)
	3	1003(19.3)	451(18.6)	1002(19.4)
	4	909(17.5)	445(18.3)	879(17.0)
	Least deprived	990(19.1)	456(18.8)	894(17.3)
Settlement	Urban	4345(83.8)	2031(83.5)	4326(83.9)
	Rural	842(16.2)	400(16.5)	830(16.1)
Strategic clinical network	Cheshire & Merseyside	257(5.0)	118(4.9)	222(4.3)
	East Midlands	463(8.9)	209(8.6)	467(9.1)
	East of England	512(9.9)	283(11.6)	578(11.2)
	Greater Manchester, Lancashire and south Cumbria	456(8.8)	233(9.6)	416(8.1)
	London	717(13.8)	383(15.8)	847(16.4)
	North East, north Cumbria, and the Hambleton & Richmondshire districts of North Yorkshire	325(6.3)	136(5.6)	281(5.4)
	South East Coast	419(8.1)	206(8.5)	424(8.2)
	South West	438(8.4)	192(7.9)	436(8.5)
	Thames Valley	172(3.3)	85(3.5)	176(3.4)
	Wessex	280(5.4)	108(4.4)	221(4.3)
	West Midlands	590(11.4)	229(9.4)	566(11.0)
	Yorkshire & The Humber	558(10.8)	249(10.2)	522(10.1)
Place of death	Hospital	2656(51.2)	1271(52.3)	2317(44.9)
	Home	2129(41.0)	896(36.9)	2069(40.1)
	Hospice	307(5.9)	212(8.7)	660(12.8)
	Elsewhere	95(1.8)	52(2.1)	110(2.1)

### Place of death and the time trends

The proportion of hospital deaths fell from over 50 % before 2005 to 45 % (95 % CI: 44–46 %) afterwards; deaths at home did not change much and fluctuated at around 40 %. The hospice deaths doubled from 4 % (95 % CI: 3–6 %) in 1993, to around 10 % (*n* = 50) up until 2008, followed by a further rise to 14 % (*n* = 73) in 2009 and remained constant at around that level since then (Fig. 1). Both the relative (0.4 % per year) and absolute (3 per year) increase of number of deaths at hospices, with the adjustment of the total number of CYP cancer deaths and CYP deaths (*p* < 0.001), were statistically significant. Most of the time hospitals were the most prevalent PoD. In 2007/2008, there was a shift in place of death: hospital deaths decreased and both home and hospice deaths increased. The percentage of hospital and home deaths were similar between 2008 and 2012, after which there was a shift to more hospital and fewer home deaths, with hospital again becoming the most prevalent place for CYP with cancer spending their last moments of life (Fig. 1).

### Spatio-temporal variations in place of death

**Fig. 2:**
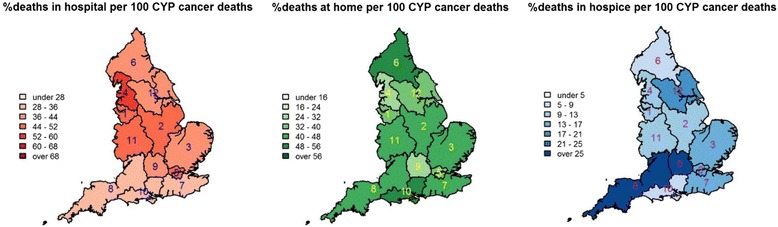
Geographical variations in place of death in children and young people who died with cancer, England 2005–2014. 1: Cheshire & Merseyside; 2: East Midlands; 3: East of England; 4: Greater Manchester, Lancashire & south Cumbria; 5: London; 6: North East, north Cumbria and the Hambleton & Richmondshire districts of North Yorkshire; 7: South East Coast; 8: South West; 9: Thames Valley; 10: Wessex; 11: West Midlands; 12: Yorkshire & the Humber

The PoD varied considerably by region, but showed an overall consistent trend for a relative reduction in hospital deaths, an increase in hospice deaths and little changes in at-home deaths from the earliest to the latest periods (Fig. 2, Appendix Figures 3 and 4). In 2005–2014 (Fig. 2), London had the highest proportion of hospital deaths (56 %, 95 % CI: 52–59 %) and lowest home deaths (31 %; 95 % CI: 28–34 %); in contrast to Wessex, where hospital deaths were the lowest (33 %; 95 % CI: 26–39 %) and home deaths the highest (56 %; 95 % CI: 49–63 %). Hospice deaths in North East, north Cumbria, and the Hambleton & Richmondshire districts of North Yorks and in Wessex (8 %; 95 % CI:5–12 %) were the lowest in the country; Thames Valley, South West and South East Coast (range:14–23 %) had the highest proportion of CYP with cancer who died in a hospice in England.

### Factors associated with place of death

Multivariable modelling results for factors associated with PoD are presented in Tables 3 and 4. In all three periods, age, CoD, number of contributing CoDs, and region were independently associated with the PoD (*P* < 0.001). Compared to young adults (20–24), those aged 0–19 years were more likely to die at home (PRs: 1.23–1.62) and less likely to die in a hospice (PRs: 0.41–0.71) than young adults, but the difference in hospice deaths was getting smaller (PRs: 0.89–0.92) and no longer significant in 2005–2014 (*P* = 0.42).

**Table 3:** Factors associated * with home deaths (versus hospital deaths) in children and young people with cancer, England 1993–2014 (*N* = 12,774)

Variable	Value	Year of death		
		1993–2000	2001–2004	2005–2014
Age(ref: 20–24)	0–14	1.62(1.44 to 1.82)	1.51(1.26 to 1.82)	1.34(1.19 to 1.50)
	15–19	1.23(1.07 to 1.41)	1.39(1.14 to 1.71)	1.28(1.12 to 1.45)
Gender	Male vs female	1.07(0.98 to 1.16)	1.04(0.91 to 1.19)	1.06(0.97 to 1.15)
Underlying cause of death (ref: Brain & CNS)	Leukaemia	0.72(0.63 to 0.82)	0.73(0.60 to 0.90)	0.58(0.51 to 0.68)
	Lymphoma & other haematology	0.75(0.61 to 0.91)	0.65(0.47 to 0.88)	0.60(0.48 to 0.74)
	Bone & connective	1.39(1.21 to 1.59)	1.29(1.06 to 1.57)	1.07(0.95 to 1.21)
	Renal/Liver/Adrenal including neuroblastoma	1.20(1.04 to 1.39)	1.27(1.02 to 1.57)	0.93(0.80 to 1.07)
	Other malignant	1.08(0.94 to 1.24)	0.90(0.71 to 1.14)	0.90(0.78 to 1.03)
	Non-cancer	0.31(0.19 to 0.49)	0.33(0.15 to 0.75)	0.57(0.37 to 0.87)
N. contributing CoDs (ref: 0)	1	0.82(0.75 to 0.91)	0.81(0.70 to 0.94)	0.82(0.74 to 0.91)
	2	0.48(0.40 to 0.57)	0.33(0.24 to 0.45)	0.40(0.33 to 0.48)
	3+	0.26(0.19 to 0.36)	0.18(0.11 to 0.29)	0.18(0.13 to 0.25)
Deprivation (ref: 5 least deprived)	1(most deprived)	0.85(0.74 to 0.97)	0.77(0.61 to 0.96)	0.76(0.66 to 0.88)
	2	0.90(0.78 to 1.03)	0.97(0.78 to 1.20)	0.83(0.72 to 0.96)
	3	0.95(0.84 to 1.09)	1.02(0.82 to 1.25)	1.00(0.87 to 1.14)
	4	0.97(0.84 to 1.11)	1.09(0.89 to 1.34)	0.99(0.86 to 1.13)
Rural/urban indicator	Rural vs Urban	1.07(0.95 to 1.20)	1.08(0.90 to 1.29)	1.00(0.89 to 1.13)
SCN region (ref: London)	Cheshire & Merseyside	1.17(0.93 to 1.48)	1.19(0.84 to 1.67)	1.01(0.78 to 1.30)
	East Midlands	1.03(0.84 to 1.25)	1.03(0.77 to 1.38)	1.09(0.90 to 1.32)
	East of England	1.27(1.06 to 1.52)	1.07(0.82 to 1.41)	1.26(1.06 to 1.51)
	Greater Manchester, Lancashire and south Cumbria	1.10(0.90 to 1.34)	0.94(0.69 to 1.27)	1.07(0.87 to 1.32)
	North East, north Cumbria, and the Hambleton & Richmondshire districts of North Yorkshire	1.23(1.01 to 1.52)	1.42(1.05 to 1.93)	1.41(1.14 to 1.73)
	South East Coast	1.06(0.87 to 1.30)	1.21(0.91 to 1.61)	1.26(1.04 to 1.53)
	South West	1.24(1.02 to 1.50)	1.03(0.76 to 1.41)	1.28(1.06 to 1.56)
	Thames Valley	1.21(0.93 to 1.57)	1.14(0.77 to 1.68)	1.09(0.82 to 1.45)
	Wessex	1.25(1.01 to 1.54)	1.17(0.84 to 1.64)	1.43(1.15 to 1.78)
	West Midlands	1.18(0.99 to 1.41)	0.86(0.63 to 1.17)	1.23(1.03 to 1.48)
	Yorkshire & The Humber	0.94(0.78 to 1.14)	1.08(0.82 to 1.44)	1.12(0.93 to 1.35)

*The association is measured by proportion ratios(PRs) and 95 % confidence intervals. PR > 1 indicates a higher probability of home deaths, <1 lower chance of home deaths, PR = 1 indicates no association. The PRs were derived from modified Poisson regression model, adjusting for the listed variables, the number of CYP deaths and the number of CYP cancer deaths at SCN region level

**Table 4:** Factors associated * with hospice deaths (versus hospital deaths) in children and young people with cancer, England 1993–2014(*N* = 12,774)

Variable	Value	Year of death		
		1993–2000	2001–2004	2005–2014
Age(ref: 20–24)	0–14	0.41(0.31 to 0.54)	0.71(0.51 to 0.98)	0.91(0.76 to 1.10)
	15–19	0.56(0.40 to 0.76)	0.63(0.42 to 0.93)	0.92(0.74 to 1.14)
Gender	Male vs female	0.86(0.68 to 1.07)	0.86(0.65 to 1.13)	0.92(0.79 to 1.08)
Underlying cause of death (ref: Brain & CNS)	Leukaemia	0.26(0.18 to 0.39)	0.35(0.22 to 0.57)	0.26(0.19 to 0.35)
	Lymphoma & other haematology	0.29(0.17 to 0.48)	0.18(0.08 to 0.43)	0.36(0.24 to 0.53)
	Bone & connective	0.93(0.65 to 1.32)	0.98(0.66 to 1.44)	0.79(0.63 to 1.00)
	Renal/Liver/Adrenal including neuroblastoma	0.76(0.46 to 1.24)	0.86(0.49 to 1.48)	0.53(0.39 to 0.73)
	Other malignant	0.77(0.57 to 1.05)	0.79(0.53 to 1.17)	0.82(0.67 to 1.02)
	Non-cancer	0.21(0.06 to 0.67)	0.14(0.02 to 1.03)	0.11(0.03 to 0.46)
N. contributing CoDs (ref: 0)	1	0.81(0.64 to 1.03)	0.53(0.37 to 0.75)	0.62(0.51 to 0.75)
	2	0.29(0.17 to 0.49)	0.25(0.13 to 0.46)	0.22(0.15 to 0.32)
	3+	0.04(0.01 to 0.26)	0.15(0.06 to 0.36)	0.16(0.10 to 0.26)
Deprivation (ref: 5 least deprived)	1(most deprived)	0.99(0.71 to 1.39)	0.82(0.52 to 1.29)	0.91(0.71 to 1.18)
	2	0.92(0.64 to 1.31)	1.06(0.68 to 1.64)	0.84(0.65 to 1.09)
	3	0.72(0.49 to 1.05)	1.15(0.74 to 1.78)	1.00(0.78 to 1.29)
	4	0.76(0.52 to 1.12)	0.97(0.61 to 1.55)	0.92(0.70 to 1.20)
Rural/Urban indicator	Rural vs Urban	0.99(0.70 to 1.40)	1.08(0.73 to 1.60)	0.96(0.77 to 1.21)
SCN region (ref: London)	Cheshire & Merseyside	2.06(1.10 to 3.85)	1.18(0.55 to 2.52)	0.91(0.59 to 1.41)
	East Midlands	0.98(0.51 to 1.90)	0.69(0.33 to 1.42)	0.97(0.69 to 1.38)
	East of England	1.51(0.84 to 2.73)	1.57(0.91 to 2.69)	1.24(0.91 to 1.70)
	Greater Manchester, Lancashire and south Cumbria	3.43(2.12 to 5.58)	1.55(0.92 to 2.63)	1.06(0.76 to 1.48)
	North East, north Cumbria, and the Hambleton & Richmondshire districts of North Yorkshire	1.04(0.48 to 2.26)	0.66(0.23 to 1.90)	0.96(0.60 to 1.54)
	South East Coast	2.46(1.42 to 4.27)	1.62(0.89 to 2.97)	1.38(0.99 to 1.92)
	South West	2.27(1.30 to 3.97)	1.38(0.75 to 2.53)	1.83(1.35 to 2.47)
	Thames Valley	1.74(0.77 to 3.93)	0.85(0.34 to 2.10)	1.71(1.18 to 2.48)
	Wessex	1.22(0.58 to 2.59)	0.52(0.18 to 1.53)	0.99(0.59 to 1.68)
	West Midlands	2.05(1.22 to 3.42)	0.88(0.48 to 1.61)	1.13(0.82 to 1.54)
	Yorkshire & The Humber	2.44(1.49 to 4.02)	1.75(1.03 to 2.98)	1.14(0.83 to 1.56)

*The association is measured by proportion ratios(PRs) and 95 % confidence intervals. PR > 1 indicates a higher probability of hospice deaths, <1 lower chance of hospice deaths, PR = 1 indicates no association. The PRs were derived from modified Poisson regression model, adjusting for the listed variables, the number of CYP deaths and the number of CYP cancer deaths at SCN region level

Both underlying cause of death (CoD) and the number of contributing CoDs were strong determinants of PoD. Compared to those who died from Brain and CNS tumours, patients with a haematological cancer or non-cancer as a underlying CoD had a significantly lower chance of dying at home (haematological cancer PRs:0.58–0.75; non-cancer PRs:0.31–0.57) or at a hospice (haematological cancer PRs:0.18–0.36; non-cancer PRs:0.11–0.21). The likelihood of death at home or in a hospice was inversely associated with the number of contributing CoDs. The chance of home death in CYPs with two or more contributing CoDs was less than half of that for those with no contributing CoD (PRs 0.18–0.48); the effect of contributing CoDs was more pronounced for hospice death (PRs:0.04–0.29). The inequality in PoD by CoD or by contributing CoDs showed no sign of narrowing down.

Residents of the two most deprived quintile areas had a reduced chance of death at home (PRs: 0.76–0.90); no disparities in home deaths were observed from the third to the highest quintile. The gap of the home deaths between the deprivation quintiles slightly widened during the study period. The chance of hospice death did not vary with deprivation. The geographical variation was persistent after controlling the potential confounding variables. Gender and type of settlement (rural/urban) were not related to where a CYP with cancer died.

There was significant interaction between the CoD and the number of CoDs in home death models (*p* < 0.001) but the condition index (CI) did not indicate the presence of significant collinearity (intercept adjusted CIs range: 1.11–1.17). However, the PR estimations from the sensitivity analyses with one of these two factors omitted from the models were only slightly different from those from the main analysis (Appendix Tables 5, 6, 7, 8).

## Discussion

This is the first population-based evaluation of the PoD and its determinants in CYP in the context of national EoLC policies and initiatives. We found that despite a 36 % reduction in CYP cancer mortality, the home death rate barely changed in the past two decades. In England, palliative care for children is typically provided at home, with support from a hospital-based oncology team coordinated by paediatric oncology outreach nurse specialists (POONS) [30]. This care model is effective in enabling home death [17, 31]. In theory, a fallen mortality may free up some home care resources to support more home deaths. However, the benefit appeared not to be transferred. A study from Taiwan using administrative data found that the EoLC in paediatric cancer patients was aggressive with heavy use of life sustaining and curative treatments, and lack of support of deaths at home [32]. Future studies need to elucidate the roles of these factors in where children and young people die. Compared to adults, the PoD change and the reduction in inequality in CYP appeared to be of a lesser degree with a time delay (e.g., until 2008). A recent study using Medicare data demonstrated that the EoLC needs in the young population may be different from those of adults [33]. Our data suggests the inadequacy of adult focused policy interventions for CYP, highlights a need for CYP-specific national initiatives that improve PEoLC care support at home.

Hospitals remained the most common PoD for CYP with cancer, around nearly half of the deaths occurred there. There was a tendency of PoD shifting from hospitals towards hospices. The shifting coincided with the landmark launch of the national EoLC programme in Nov 2004 and subsequent national efforts [13]. The PoD in adults with cancer during the same period in England shifted towards both home and hospices with more pronounced effects on home deaths [17]. The PoD shifting outside hospitals is consistent with findings from other countries [34–36]. The hospice deaths increased but were still rather low in most England’s regions, ranging from under 10 % to around 20 %. Hospices have been mostly used as a respite service in the UK. Their potential as a great alternative to hospital in meeting the needs of managing physical symptoms has not yet been fully exploited. Future research needs to focus on how to increase and expand the use of hospice services.

Although the chance of home and hospice deaths for CYPs with most cancers improved though slowly over time, deaths of those with haematological cancer still predominantly (~70 %) took place in hospitals. There was little change in this proportion over time, similar to what’s seen in adults [17]. Haematological cancers accounted for nearly 1 in 3 CYP cancer deaths. Over half of the non-cancer deaths had a haematological cancer as a contributing CoD. This is consistent with a previous systematic review and meta-analysis [37]. The review also highlighted that the reasons for the high hospital deaths in haematology patients are not yet clear, but likely to be multidimensional and complex. The number of CoDs has been used as a proxy measure of comorbidities [38, 39], which, in turn, translates into the care management complexity at the end of life [40]. The CYP deaths with more contributing CoDs were less likely to occur at home or in a hospice. While these results should be interpreted with caution as the recording practice may vary by location of death, constantly improving cancer survival and increasing comorbidities among cancer survivors are bound to make EoLC management more challenging [41, 42].

Given that home and hospices are preferred and increasingly important for CYP cancer patients to spend their last moments of life, the healthcare system should be better equipped to meet such needs. Surprisingly, compared to those with no contributing CoDs, a patient with two or more contributing CoDs had 80 % lower chance of dying in a hospice, even lower than that of home deaths. It appears that when it comes to cases with comorbid conditions, hospice as a dedicated EoLC facility does not seem to be more advantageous than people’s own home. Further investigations to the reasons are needed. The level of deprivation was related to lower chances of home deaths but not hospice deaths, the inequality gap showed little sign of closing up. The wide regional variations in PoD are worth exploring in depth, as a better understanding of these variations may reveal important clues for local practices, policies and service configurations that facilitate and enable home or hospice death.

It is worth noting that these findings were observed in the background of a relative increase (from 18 to 25 %) in CYP cancer deaths, partly due to the mortality reduction in competing causes of death (e.g., diabetes, asthma) [43]. This trend is likely to continue, given the rise of the cancer incidence [44]. Our findings suggest that even in the traditionally and relatively better served disease group – cancer [45], the end of life care for CYP is suboptimal. It is even more unfortunate, as it happened in a country ranked consistently the best in the world for its quality of death [46]. There is a pressing need for national and international actions to improve end of life care for this neglected CYP population.

This study has several limitations. We did not have information regarding care and transition of the care settings; the death certificate data only contains an individual’s final PoD. Even if a patient was admitted to a hospital in the last minute and died there, the PoD will be recorded as the hospital. There is evidence that preferred PoD may differ from preferred place of care^25^. We should also note that we did not have information on preferences of the patient and family members, or indicators for clinical appropriateness of PoD. Nevertheless, as a patient and/or their carer’s preference for where to die is highly dependent on the level of care support one can get in a specific setting [2, 47], the PoD is still a useful indicator for EoLC needs and to what extent the need has been met.

## Conclusions

Hospitals and home were the main EoLC setting for CYP with cancer. The home death rate (~40 %) barely changed in the past two decades, and deaths in hospital remained the most common but slightly shifted towards hospices. CYP with haematological malignancy and with comorbid conditions had persistently high hospital deaths; the chance of these cases with deaths in hospices was even lower than at home. There were deprivation- and area-related inequalities in PoD which may need service-level change and/or policy-level intervention. The findings highlight a need for CYP specific initiatives to enhance EoLC support and capacities at home and in hospices.

## Figures and Tables

**Fig. 3:**
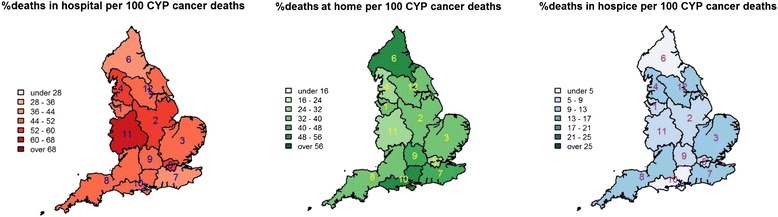
Geographical variations in place of death in children and young people who died with cancer, England 2001─2004. 1: Cheshire & Merseyside; 2: East Midlands; 3: East of England; 4: Greater Manchester, Lancashire & South Cumbria; 5: London; 6: North East, North Cumbria and the Hambleton & Richmondshire districts of North Yorkshire; 7: South East Coast; 8: South West; 9: Thames Valley; 10: Wessex; 11: West Midlands; 12: Yorkshire & the Humber

**Fig. 4:**
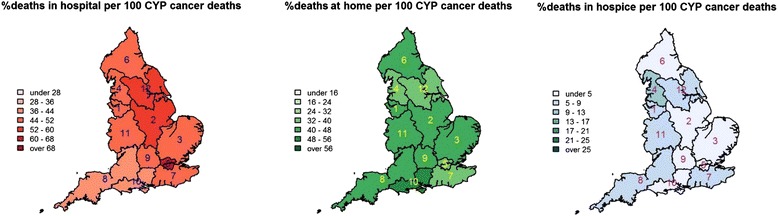
Geographical variations in place of death in children and young people who died with cancer, England 1993─2000. 1: Cheshire & Merseyside; 2: East Midlands; 3: East of England; 4: Greater Manchester, Lancashire & South Cumbria; 5: London; 6: North East, North Cumbria and the Hambleton & Richmondshire districts of North Yorkshire; 7: South East Coast; 8: South West; 9: Thames Valley; 10: Wessex; 11: West Midlands; 12: Yorkshire & the Humber

## Appendix

**Table 5:** Factors associated * with home death(versus hospital death) in children and young people with cancer, England 1993–2014(*N* = 12,774)

Variable	Value	Year of death		
		1993–2000	2001–2004	2005–2014
Age(ref: 20–24)	0–14	1.64(1.46 to 1.84)	1.47(1.23 to 1.77)	1.34(1.19 to 1.50)
	15–19	1.23(1.07 to 1.42)	1.33(1.09 to 1.63)	1.24(1.10 to 1.41)
Gender	Male vs female	1.07(0.98 to 1.17)	1.06(0.93 to 1.21)	1.06(0.98 to 1.16)
Underlying cause of death (ref: Brain & CNS)	Leukaemia	0.65(0.57 to 0.74)	0.60(0.49 to 0.74)	0.47(0.41 to 0.55)
	Lymphoma & other haematology	0.71(0.58 to 0.86)	0.59(0.44 to 0.81)	0.52(0.42 to 0.65)
	Bone & connective	1.33(1.16 to 1.52)	1.27(1.04 to 1.54)	1.08(0.96 to 1.23)
	Renal/Liver/Adrenal including neuroblastoma	1.18(1.02 to 1.36)	1.31(1.06 to 1.62)	0.92(0.79 to 1.06)
	Other malignant	1.02(0.89 to 1.17)	0.79(0.63 to 1.00)	0.83(0.72 to 0.95)
	Non-cancer	0.18(0.11 to 0.28)	0.15(0.07 to 0.34)	0.26(0.18 to 0.40)
Deprivation (ref: 5 least deprived)	1(most deprived)	0.83(0.73 to 0.96)	0.78(0.62 to 0.97)	0.72(0.63 to 0.84)
	2	0.88(0.76 to 1.01)	0.98(0.79 to 1.21)	0.81(0.70 to 0.93)
	3	0.94(0.83 to 1.08)	1.02(0.82 to 1.25)	0.98(0.85 to 1.12)
	4	0.95(0.83 to 1.08)	1.12(0.91 to 1.37)	0.99(0.87 to 1.14)
Rural/urban indicator	Rural vs Urban	1.07(0.95 to 1.20)	1.08(0.90 to 1.28)	1.03(0.91 to 1.15)
SCN region (ref: London)	Cheshire & Merseyside	1.19(0.95 to 1.50)	1.27(0.90 to 1.79)	1.04(0.81 to 1.34)
	East Midlands	1.07(0.88 to 1.30)	1.10(0.82 to 1.47)	1.12(0.92 to 1.35)
	East of England	1.30(1.08 to 1.56)	1.08(0.82 to 1.42)	1.26(1.05 to 1.50)
	Greater Manchester, Lancashire and south Cumbria	1.15(0.95 to 1.41)	0.96(0.71 to 1.30)	1.07(0.87 to 1.31)
	North East, north Cumbria, and the Hambleton & Richmondshire districts of North Yorkshire	1.29(1.05 to 1.59)	1.41(1.04 to 1.91)	1.48(1.20 to 1.83)
	South East Coast	1.11(0.91 to 1.36)	1.22(0.92 to 1.62)	1.27(1.05 to 1.54)
	South West	1.30(1.07 to 1.57)	1.04(0.77 to 1.42)	1.30(1.07 to 1.58)
	Thames Valley	1.28(0.99 to 1.66)	1.15(0.78 to 1.69)	1.11(0.84 to 1.48)
	Wessex	1.35(1.09 to 1.67)	1.19(0.85 to 1.66)	1.48(1.19 to 1.84)
	West Midlands	1.21(1.01 to 1.44)	0.84(0.61 to 1.14)	1.22(1.02 to 1.46)
	Yorkshire & The Humber	0.94(0.78 to 1.15)	1.07(0.81 to 1.42)	1.17(0.97 to 1.41)

*The association is measured by proportion ratios(PRs) and 95 % confidence intervals. PR > 1 indicates a higher probability of home death, <1 lower chance of home death, PR = 1 indicates no association. The PRs were derived from modified Poisson regression model, adjusting for the listed variables in the main analysis but with the **omission of the number of contributing CoDs**, the number of CYP deaths and the number of CYP cancer deaths at SCN region level

**Table 6:** Factors associated * with home death(versus hospital death) in children and young people with cancer, England 1993–2014(*N* = 12,774)

Variable	Value	Year of death		
		1993–2000	2001–2004	2005–2014
Age(ref: 20–24)	0–14	1.62(1.45 to 1.80)	1.58(1.33 to 1.88)	1.37(1.23 to 1.53)
	15–19	1.22(1.06 to 1.40)	1.45(1.19 to 1.78)	1.30(1.15 to 1.48)
Gender	Male vs female	1.05(0.97 to 1.15)	1.01(0.89 to 1.16)	1.04(0.96 to 1.14)
N. contributing CoDs (ref: 0)	1	0.85(0.77 to 0.93)	0.80(0.69 to 0.93)	0.81(0.73 to 0.89)
	2	0.42(0.35 to 0.50)	0.28(0.21 to 0.38)	0.35(0.29 to 0.42)
	3+	0.20(0.15 to 0.28)	0.15(0.09 to 0.24)	0.15(0.11 to 0.20)
Deprivation (ref: 5 least deprived)	1(most deprived)	0.85(0.74 to 0.97)	0.77(0.62 to 0.97)	0.75(0.65 to 0.87)
	2	0.89(0.78 to 1.02)	0.99(0.80 to 1.22)	0.82(0.71 to 0.95)
	3	0.94(0.82 to 1.07)	1.05(0.85 to 1.30)	0.98(0.86 to 1.12)
	4	0.96(0.84 to 1.10)	1.09(0.89 to 1.33)	0.99(0.86 to 1.13)
Rural/Urban indicator	Rural vs Urban	1.07(0.95 to 1.20)	1.10(0.92 to 1.31)	1.01(0.89 to 1.13)
SCN region (ref: London)	Cheshire & Merseyside	1.18(0.94 to 1.49)	1.14(0.81 to 1.60)	1.01(0.78 to 1.29)
	East Midlands	1.06(0.87 to 1.28)	1.03(0.77 to 1.38)	1.08(0.90 to 1.31)
	East of England	1.29(1.08 to 1.55)	1.14(0.87 to 1.50)	1.26(1.05 to 1.51)
	Greater Manchester, Lancashire and south Cumbria	1.12(0.91 to 1.36)	0.97(0.72 to 1.31)	1.05(0.86 to 1.29)
	North East, north Cumbria, and the Hambleton & Richmondshire districts of North Yorkshire	1.23(1.00 to 1.51)	1.51(1.11 to 2.04)	1.41(1.14 to 1.73)
	South East Coast	1.07(0.88 to 1.32)	1.27(0.96 to 1.69)	1.27(1.05 to 1.55)
	South West	1.27(1.05 to 1.54)	1.05(0.77 to 1.43)	1.29(1.06 to 1.57)
	Thames Valley	1.20(0.93 to 1.56)	1.17(0.79 to 1.72)	1.08(0.82 to 1.44)
	Wessex	1.28(1.03 to 1.58)	1.27(0.91 to 1.77)	1.45(1.16 to 1.80)
	West Midlands	1.20(1.00 to 1.43)	0.89(0.65 to 1.20)	1.22(1.02 to 1.46)
	Yorkshire & The Humber	0.96(0.79 to 1.17)	1.12(0.84 to 1.48)	1.12(0.93 to 1.35)

*The association is measured by proportion ratios(PRs) and 95 % confidence intervals. PR > 1 indicates a higher probability of hospice death, <1 lower chance of hospice death, PR = 1 indicates no association. The PRs were derived from modified Poisson regression model, adjusting for the listed variables in the main analysis but with the **omission of the Underlying CoD**, number of CYP deaths and number of CYP cancer deaths at SCN region level. -: not estimable

**Table 7:** Factors associated * with hospice death(versus hospital death) in children and young people with cancer, England 1993–2014(*N* = 12,774)

Variable	Value	Year of death		
		1993–2000	2001–2004	2005–2014
Age (ref: 20–24)	0–14	0.37(0.28 to 0.49)	0.59(0.43 to 0.81)	0.85(0.71 to 1.02)
	15–19	0.54(0.39 to 0.74)	0.54(0.37 to 0.80)	0.82(0.66 to 1.02)
Gender	Male vs female	0.85(0.68 to 1.07)	0.89(0.68 to 1.16)	0.94(0.80 to 1.09)
Underlying cause of death (ref: Brain & CNS)	Leukaemia	0.21(0.14 to 0.31)	0.25(0.16 to 0.40)	0.17(0.13 to 0.24)
	Lymphoma & other haematology	0.26(0.16 to 0.43)	0.16(0.07 to 0.36)	0.29(0.20 to 0.43)
	Bone & connective	0.86(0.61 to 1.21)	0.96(0.65 to 1.41)	0.80(0.64 to 1.01)
	Renal/Liver/Adrenal including neuroblastoma	0.71(0.43 to 1.16)	0.85(0.49 to 1.48)	0.51(0.37 to 0.70)
	Other malignant	0.72(0.53 to 0.97)	0.68(0.46 to 1.00)	0.73(0.59 to 0.90)
	Non-cancer	0.09(0.03 to 0.28)	0.05(0.01 to 0.39)	0.04(0.01 to 0.16)
Deprivation (ref: 5 least deprived)	1(most deprived)	0.99(0.71 to 1.39)	0.94(0.60 to 1.48)	0.88(0.68 to 1.13)
	2	0.89(0.62 to 1.28)	1.15(0.74 to 1.77)	0.82(0.64 to 1.07)
	3	0.70(0.48 to 1.02)	1.18(0.76 to 1.84)	0.97(0.75 to 1.25)
	4	0.73(0.50 to 1.07)	1.03(0.65 to 1.64)	0.92(0.70 to 1.20)
Rural/urban indicator	Rural vs Urban	0.99(0.70 to 1.39)	1.11(0.75 to 1.64)	0.99(0.78 to 1.24)
SCN region (ref: London)	Cheshire & Merseyside	2.09(1.12 to 3.91)	1.27(0.59 to 2.72)	0.99(0.64 to 1.53)
	East Midlands	1.01(0.52 to 1.95)	0.78(0.38 to 1.61)	1.00(0.71 to 1.42)
	East of England	1.58(0.88 to 2.86)	1.66(0.97 to 2.86)	1.22(0.89 to 1.67)
	Greater Manchester, Lancashire and south Cumbria	3.57(2.20 to 5.81)	1.64(0.97 to 2.78)	1.08(0.78 to 1.52)
	North East, north Cumbria, and the Hambleton & Richmondshire districts of North Yorkshire	1.07(0.49 to 2.31)	0.59(0.21 to 1.71)	1.03(0.64 to 1.65)
	South East Coast	2.60(1.50 to 4.50)	1.57(0.86 to 2.86)	1.39(1.00 to 1.94)
	South West	2.34(1.34 to 4.10)	1.40(0.76 to 2.59)	1.81(1.34 to 2.45)
	Thames Valley	1.84(0.82 to 4.15)	0.99(0.40 to 2.45)	1.81(1.25 to 2.62)
	Wessex	1.36(0.64 to 2.88)	0.52(0.18 to 1.51)	0.99(0.58 to 1.67)
	West Midlands	2.09(1.25 to 3.49)	0.87(0.47 to 1.59)	1.12(0.82 to 1.54)
	Yorkshire & The Humber	2.40(1.46 to 3.94)	1.83(1.08 to 3.11)	1.24(0.91 to 1.69)

*The association is measured by proportion ratios(PRs) and 95 % confidence intervals. PR > 1 indicates a higher probability of home death, <1 lower chance of home death, PR = 1 indicates no association. The PRs were derived from modified Poisson regression model, adjusting for the listed variables in the main analysis but with the **omission of the number of contributing CoDs**, the number of CYP deaths and the number of CYP cancer deaths at SCN region level

**Table 8:** Factors associated * with hospice death(versus hospital death) in children and young people with cancer, England 1993–2014(*N* = 12,774)

Variable	Value	Year of death		
		1993–2000	2001–2004	2005–2014
Age(ref: 20–24)	0–14	0.44(0.34 to 0.58)	0.77(0.57 to 1.05)	0.95(0.80 to 1.12)
	15–19	0.52(0.38 to 0.71)	0.62(0.42 to 0.92)	0.88(0.71 to 1.10)
Gender	Male vs female	0.82(0.65 to 1.03)	0.81(0.62 to 1.07)	0.92(0.79 to 1.07)
N. contributing CoDs(ref: 0)	1	0.77(0.61 to 0.98)	0.46(0.33 to 0.65)	0.56(0.46 to 0.67)
	2	0.22(0.13 to 0.37)	0.18(0.10 to 0.33)	0.15(0.10 to 0.23)
	3+	0.02(0.00 to 0.17)	0.10(0.04 to 0.24)	0.10(0.06 to 0.17)
Deprivation(ref: 5 least deprived)	1(most deprived)	1.06(0.76 to 1.48)	0.83(0.53 to 1.30)	0.90(0.70 to 1.16)
	2	0.92(0.64 to 1.32)	1.07(0.69 to 1.65)	0.85(0.66 to 1.10)
	3	0.72(0.49 to 1.05)	1.17(0.75 to 1.81)	1.03(0.80 to 1.32)
	4	0.77(0.52 to 1.13)	1.03(0.65 to 1.64)	0.90(0.69 to 1.18)
Rural/Urban indicator	Rural vs Urban	1.02(0.73 to 1.45)	1.11(0.76 to 1.64)	0.97(0.77 to 1.22)
SCN region (ref: London)	Cheshire & Merseyside	2.02(1.08 to 3.78)	1.12(0.52 to 2.39)	0.86(0.55 to 1.32)
	East Midlands	1.02(0.53 to 1.96)	0.69(0.34 to 1.42)	0.90(0.64 to 1.28)
	East of England	1.55(0.86 to 2.79)	1.59(0.93 to 2.73)	1.23(0.90 to 1.68)
	Greater Manchester, Lancashire and south Cumbria	3.35(2.07 to 5.44)	1.64(0.97 to 2.77)	1.05(0.75 to 1.47)
	North East, north Cumbria, and the Hambleton & Richmondshire districts of North Yorkshire	0.94(0.43 to 2.04)	0.63(0.22 to 1.81)	0.88(0.55 to 1.42)
	South East Coast	2.46(1.42 to 4.26)	1.66(0.91 to 3.03)	1.43(1.03 to 1.99)
	South West	2.51(1.44 to 4.39)	1.43(0.77 to 2.62)	1.78(1.31 to 2.41)
	Thames Valley	1.56(0.69 to 3.51)	0.95(0.39 to 2.34)	1.70(1.17 to 2.47)
	Wessex	1.37(0.65 to 2.89)	0.59(0.20 to 1.71)	0.99(0.58 to 1.67)
	West Midlands	2.04(1.22 to 3.41)	0.95(0.52 to 1.74)	1.11(0.81 to 1.52)
	Yorkshire & The Humber	2.60(1.59 to 4.27)	1.78(1.05 to 3.00)	1.09(0.80 to 1.48)

*The association is measured by proportion ratios(PRs) and 95 % confidence intervals. PR > 1 indicates a higher probability of hospice death, <1 lower chance of hospice death, PR = 1 indicates no association. The PRs were derived from modified Poisson regression model, adjusting for the listed variables in the main analysis but with the **omission of the Underlying CoD**, number of CYP deaths and number of CYP cancer deaths at SCN region level. -: not estimable

## Abbreviations

CYP: Children and young people
ONS: Office for National Statistics
PEoLC: Palliative and end of life care
PoD: Place of death
